# Oxidative Stress in COPD: Sources, Markers, and Potential Mechanisms

**DOI:** 10.3390/jcm6020021

**Published:** 2017-02-15

**Authors:** Adam John Anthony McGuinness, Elizabeth Sapey

**Affiliations:** Institute of Inflammation and Ageing, University of Birmingham, Birmingham B15 2TT, UK; sapeye@adf.bham.ac.uk

**Keywords:** COPD, oxidative stress, neutrophil, macrophage, antioxidant, antiproteinase, mechanisms, therapeutic studies

## Abstract

Markers of oxidative stress are increased in chronic obstructive pulmonary disease (COPD) and reactive oxygen species (ROS) are able to alter biological molecules, signaling pathways and antioxidant molecule function, many of which have been implicated in the pathogenesis of COPD. However, the involvement of ROS in the development and progression of COPD is not proven. Here, we discuss the sources of ROS, and the defences that have evolved to protect against their harmful effects. We address the role that ROS may have in the development and progression of COPD, as well as current therapeutic attempts at limiting the damage they cause. Evidence has indicated that the function of several key cells appears altered in COPD patients, and expression levels of important oxidant and antioxidant molecules may be abnormal. Therapeutic trials attempting to restore equilibrium to these molecules have not impacted upon all facets of disease and whilst the theory behind ROS influence in COPD appears sound, current models testing relevant pathways to tissue damage are limited. The heterogeneity seen in COPD patients presents a challenge to our understanding, and further research is essential to identify potential targets and stratified COPD patient populations where ROS therapies may be maximally efficacious.

## 1. Introduction

Chronic obstructive pulmonary disease (COPD) is a chronic respiratory lung condition with significant systemic manifestations and associated co-morbidities that deleteriously impacts on quality of life [1]. It is characterised by airflow obstruction and associated with lung inflammation and tissue destruction. COPD is a disease of ageing; in the UK, the prevalence of diagnosed COPD in the 45–54 age group is <1%, rising to >5% in over 65s, however, this is likely to be an under-representation of COPD prevalence due to under diagnosis [2]. COPD is currently responsible for one out of eight emergency admissions to hospital, with a total cost of more than £800 m per year [3], and is currently the fourth leading cause of death worldwide, predicted to become the third by 2030 [4].

Smoking and air pollution have been identified as significant initiating and risk factors for COPD and cell damage and death seen in COPD airways has been attributed to oxidative and carbonyl stress [5]. Only 15%–20% of smokers develop COPD and cessation of smoking does not halt progression of the disease, with continued evidence of inflammatory cell recruitment to the lungs (in particular neutrophil recruitment) and oxidative stress [6]. This indicates a self-perpetuating endogenous source of inflammation in susceptible individuals [7,8]. Continued release of inflammatory mediators such as leukotriene B4 (LTB4) and interleukin (IL)-8 [9] amongst others, encourages continued recruitment and activation of neutrophils to the lungs. The release of proteases, free radicals and cytokines from these activated cells has been implicated in all facets of COPD including the destruction of surrounding tissues, a loss of lung elasticity [10] and mucus hypersecretion [11]; associated with emphysema and chronic bronchitis in COPD.

Oxidative stress occurs when exposure to free radicals is sufficient to overwhelm antioxidant defences. Such free radicals, termed reactive oxygen species (ROS), are ubiquitous, arising during mitochondrial respiration, signaling and when contributing to the damage and destruction of pathogens. Common ROS include hydroxyl radical (·OH) and superoxide anion (O_2_·^−^) which contain unpaired electrons, the unstable nature of these ROS permit transfer of electrons to other molecules via oxidation, resulting in damage, inactivation or creation of further ROS. Potential targets for damage by ROS include proteins, lipids or DNA. Lungs are particularly vulnerable to oxidative stress due to the relatively high oxygen environment, increased blood supply and exposure to environmental pathogens and toxins. Cigarette and biomass smoke add significantly to this burden, a single puff of cigarette smoke is estimated to have in excess of 1 × 10^15^ oxidant molecules within [12].

There is significant theoretical support for the hypothesis that ROS will damage the lungs and contribute to the pathogenesis of COPD, but here we examine the evidence for such a relationship. This review will discuss the formation and roles of ROS in humans and the defences that have evolved to mitigate collateral damage. We present evidence of the association between oxidative stress and COPD, and attempts to replicate the mechanisms that may be involved. Evidence for pro- and anti-inflammatory imbalances that may lie at the heart of ROS involvement in COPD, and attempts to mitigate these via therapeutic treatments are also discussed.

## 2. Production of ROS

ROS generation has two main roles, as currently understood; bactericidal activity and intracellular signalling.

Antimicrobial ROS production in phagocytic cells relies upon nicotinamide adenine dinucleotide phosphate(NADPH)-oxidase (NOX2). NOX2 consists of multiple subunits, spatially separated and inactivated in a resting phagocyte in order to prevent ROS generation in the quiescent state. Subunits gp91^ph^°^x^ and p22^ph^°^x^ are co-located integral membrane proteins, and together form flavocytochrome b_558_ (cyt b_558_), whilst subunits p40^ph^°^x^, p47^ph^°^x^, p67^ph^°^x^ exist as a complex in the cytosol. Activation of a phagocyte via pattern recognition receptors (PRRs) on the cell surface, such as toll-like receptors, results in nuclear factor kappa-light-chain-enhancer of activated B cells (NF-κB) signalling and subsequent phosphorylation of p47^ph^°^x^, initiating translocation of the cytosolic complex to the membrane. Activation of NOX2 is completed by the co-localisation of two final proteins, Rac2 and Rap1A. Upon activation, cytosolic Rac2 binds guanosine triphosphate and translocates to the membrane with p40^ph^°^x^, p47^ph^°^x^ and p67^ph^°^x^, whereupon it associates with cyt b_558_ and Rap1A. As the process of activation of PRRs also initiates phagocytic envelopment of the microbes, and fusion with a lysosome, the membrane localisation of the assembled NOX2 protein becomes the membrane of the phagolysosome. Active NOX2 transfers an electron from NADPH to O_2_ resulting in the formation of superoxide anion (O_2_·^−^) which is released into the phagolysosome [13]. This process is reflected in the Equation (1) shown below:

NADPH + 2O_2_ → NADP^+^ + 2 O_2_·^−^ + H^+^ (1)

The superoxide anion is unstable due to the absence of a hydrogen ion and resolving this state is energetically favourable, resulting in a willingness of superoxide to bind to other substances in order to share electrons. Direct oxidation via superoxide is considered to contribute little to oxidative stress, in particular because of the rapid dismutation of superoxide into hydrogen peroxide (H_2_O_2_), catalysed by the enzyme superoxide dismutase (SOD) in the following Reaction (2):

O_2_·^−^ + O_2_·^−^ + 2H^+^ → H_2_O_2_ + O_2_ (2)

A further species of ROS common in phagocytes is nitric oxide (·NO), this is readily produced via inducible nitric oxide synthase following the stimulation of phagocytes by pathogen associated molecular patterns (PAMPs) such as lipopolysaccharide (LPS) and tumour necrosis factor (TNF)α [14]. It is also removed extremely quickly in vivo, typically by diffusion into the blood stream where it is scavenged by oxyhaemoglobin in red blood cells. Rapid removal of ·NO and O_2_·^−^ is essential in health, as when the two are at increased concentrations, as seen in inflammation, they combine extremely readily to form peroxynitrite (ONOO^−^). Peroxynitrite is an extremely damaging molecule thanks to its enhanced stability, allowing it to diffuse throughout tissues where it is capable of oxidising multiple targets, including protein thiols [15], and tyrosine [16]. 

**Figure 1:**
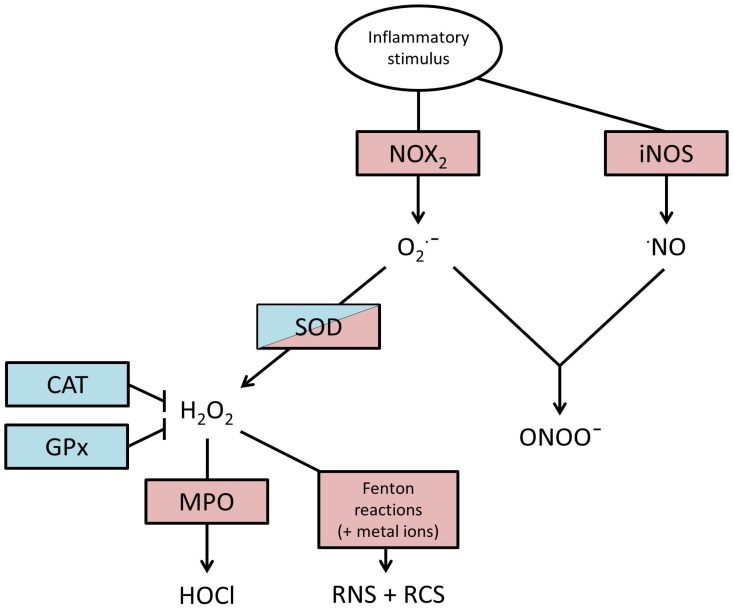
Molecular production of reactive species. Initiated by an inflammatory stimulus, a cascade begins that results in a variety of oxidative agents. Red boxes represent pro-inflammatory enzymes, whilst blue boxes represent anti-inflammatory enzymes. Superoxide dismutase (SOD) may be classed as both as its substrate and product are both capable of causing harm. NOX2, nicotinamide adenine dinucleotide phosphate(NADPH)-oxidase; iNOS, inducible nitric oxide synthase; O_2_·^−^, superoxide anion; ·NO, nitric oxide; SOD, superoxide dismutase; H_2_O_2_, hydrogen peroxide; CAT, catalase; GPx, glutathione peroxidase; MPO, myeloperoxidase; HOCl, hypochlorous acid; RCS + RNS, reactive nitrogen species + reactive carbon species; ONOO¯, peroxynitrite.

Phagocyte lysosomes also contain the enzyme myeloperoxidase [17] which catalyses H_2_O_2_, producing highly oxidising hypochlorous acid (HOCl). Furthermore, iron and other metal ions can catalyse the production of hydroxyl radicals from H_2_O_2_ via Haber–Weiss and Fenton reactions. This chain of oxidation events leads to the formation of reactive nitrogen and carbonyl species (RNS and RCS) alongside ROS, with similar damaging potential. This pathway of events and chief ROS outcomes are illustrated in Figure 1. 

Environmental derived ROS are common at the lung epithelium, found not only within cigarette smoke and combustion of organic matter [18], but also gases capable of oxidant activity such as ozone and nitrogen dioxide can deplete oxidant defences [19,20,21], increasing oxidative burden.

## 3. Actions of ROS

Reactive species and by-products are capable of altering thiols [22] and amines [23], as well as amino acid residues such as cysteine, methionine and tyrosine [24]. These alterations can lead to changed charge profiles and the formation of new disulphide bonds, altering tertiary protein structure and overall protein function [25] leading to altered activity [26]. For example, oxidation of methionine 358 in the active site of α1 antitrypsin (α1AT) results in an inability of α1AT to inhibit neutrophil elastase [27,28]. In addition to altering proteins, lipids [29,30] and DNA [31] are vulnerable to damage from ROS, and along with them, the potential for membrane dysfunction and transcriptional errors.

Production of ROS is not limited to pathogen defence. ROS are key in mitochondrial respiration as part of the electron transport chain, during which electrons are passed from donors to acceptors in the process powering the transfer of protons across membranes. Under certain conditions (reviewed elsewhere [32]), ROS can be released intracellularly, altering cellular homeostasis and transcription.

ROS are also involved in cell signalling. Typical intracellular signalling occurs on the macromolecular level, proteins interacting with proteins, ligands with receptors, with shape and surface charge being key to specificity. By their nature, ROS react indiscriminately with vulnerable atomic sequences, contrasting with the traditional view of specificity required in signalling. However, this indiscriminate, atomic level signalling allows for influence in multiple pathways simultaneously, permitting a more nuanced response from effector proteins, responsive to intracellular conditions and other pathways not directly linked via macromolecules. For example, protein tyrosine phosphatases (PTPs) can be inactivated in the presence of H_2_O_2_, which is reversible with glutathione and other thiols [33]. Via this method, the intracellular release of ROS is able to regulate tyrosine phosphorylation, and therefore kinase mediated signalling in multiple pathways. This may have relevance in COPD where the PTP domain containing protein, phosphatase and tensin homolog (PTEN) [34] plays a key role in the activation and migration of neutrophils, strongly linked with COPD pathogenesis, as discussed later.

## 4. Endogenous Defences against ROS

ROS can be inactivated by both enzymatic and non-enzymatic means, and these are present in abundance in health. Non-enzymatic antioxidant defence consists of antioxidant molecules (ascorbic acid), metal binding proteins, sacrificial proteins and unsaturated lipids, which act as electron donors or recipients. Two such antioxidant molecules found in lung epithelial lining fluid are vitamin C (ascorbate) and vitamin E (tocopherol). Tocopherol acts as an antioxidant by donating an electron from its aromatic ring to ·OH to produce H_2_O and the much less damaging vitamin E radical. Ascorbate acts by donating electrons to lipid peroxyls, or to vitamin E radical, returning them to safe states, whilst itself becoming the very stable ascorbate radical [35].

Antioxidant sacrificial proteins, such as albumin and mucin in epithelial fluid, and glutathione within cells, also function as antioxidants by the nature of their surface exposed methionine and cysteine residues. These residues are readily oxidised by a number of ROS [36,37], scavenging oxidants before they can interact with structurally or functionally important residues [38,39]. This protective function is not only limited to sacrificial proteins, but also includes sacrificial residues within functional proteins. Alpha 2-macroglobulin, a human antiproteinase, retains its function after oxidation of eight methionine residues, however, oxidation of a further six residues and a tryptophan residue resulted in loss of function [40]. Once oxidised, these residues can be recycled back to their original state by enzymes such as methionine sulfoxide reductases (particularly abundant in neutrophils [41]) and thioredoxin, replenishing the antioxidant pool.

Enzymatic antioxidants include superoxide dismutase (SOD), catalase and glutathione peroxidase (GPx). Superoxide dismutase in its three forms (SOD1, SOD2, SOD3) has very high affinity for O_2_^−^ converting it to H_2_O_2_. It functions as an antioxidant by removing superoxide anions before they have the time to cause damage, or the opportunity to interact with other radicals to produce extremely damaging peroxyl radicals. However, in this process, H_2_O_2_ is produced, itself less damaging than O_2_^−^, but H_2_O_2_ can take part in Haber–Weiss and Fenton reactions to produce ·OH hydroxyl radicals which very readily react with substrates to become hydroxyl groups (see Figure 1). The enzymes catalase and GPx have evolved to combat this threat, and both have a high affinity for H_2_O_2_, catalase converts two H_2_O_2_ molecules to two H_2_O and O_2_. GPx catalyses the reduction of H_2_O_2_ and peroxides present on oxidised molecules, in this reaction, Glutathione (GSH) acts as a hydrogen ion donor, becoming glutathione disulphide (GSSG), the donated hydrogen ion can then inactivate the ROS molecule.

**Figure 2:**
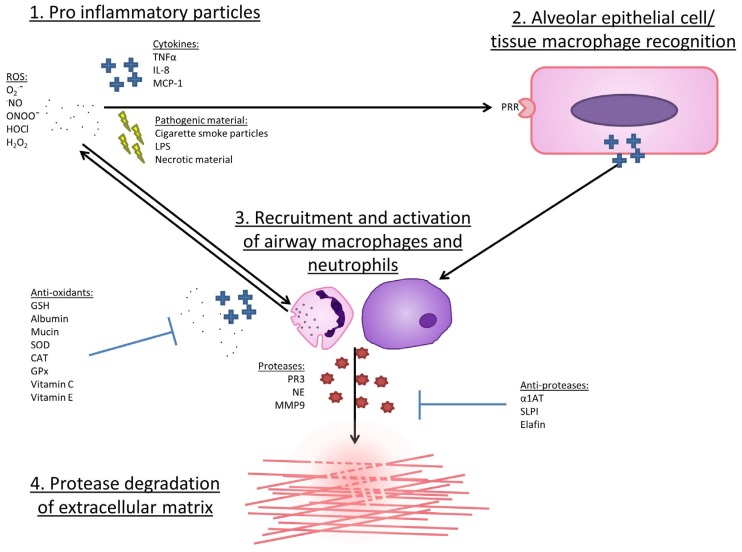
Process of tissue damage in chronic obstructive pulmonary disease (COPD). 1. Influx of pro-inflammatory particles into lung. 2. Particles bind PRRs on the surface of lung epithelial or tissue macrophages, instigating a signalling cascade culminating in the release of pro-inflammatory cytokines. 3. Cytokines encourage the recruitment, migration and activation of peripheral neutrophils and monocytes, the latter of which mature to alveolar macrophages once recruited. Activation of these cells results in the further cytokine release, proteases and ROS, in turn fueling further recruitment and activation. In health, anti-proteases and anti-oxidants are present in very high levels, preventing excess damage to surrounding tissues, and limiting a positive feedback loop. In COPD, these levels may be lacking, resulting in increased areas of obligate tissue damage. 4. If insufficiently inhibited, proteases will degrade extracellular matrix, leading to tissue destruction and pathologies seen in COPD. O_2_·¯, superoxide anion; ·NO, nitric oxide; SOD, superoxide dismutase; H_2_O_2_, hydrogen peroxide; CAT, catalase; GPx, glutathione peroxidase; MPO, myeloperoxidase; HOCl, hypochlorous acid; ONOO^−^, peroxynitrite; TNFα, tumour necrosis factor α; IL8, Interleukin 8; MCP-1, monocyte chemoattractant protein 1; LPS, lipopolysaccharide; PRR, pattern recognition receptor; GSH, glutathione; PR3, proteinase 3; NE, neutrophil elastase; MMP9, matrix metalloproteinase 9; α1AT, α1 antitrypsin; SLPI, secretory leukocyte protease inhibitor.

Antioxidant molecules are maintained at very high concentrations in vivo in order to rapidly swamp ROS that are released extracellularly. Albumin is the most abundant protein in serum, whilst glutathione levels in alveolar lining fluid is approximately 140 times higher than in blood in healthy individuals [42] and these high extracellular levels are required in order to protect against the potential for damage offered by ROS. Intracellular levels of antioxidant are more carefully restricted, in part due to the essential nature of ROS in mitochondrial respiration and signalling. The chain of events leading to ROS release and obligate tissue damage is illustrated in Figure 2.

## 5. Evidence for an Association between Oxidative Stress and COPD

A variety of methods have been employed to assess the presence of oxidative stress in the lungs of COPD patients, and there is clear evidence of an increased oxidative burden in COPD compared with non-smoking controls.

Exhaled breath condensate (EBC) is an effective method used to identify oxidative stress products found in the lungs [42]. Multiple studies have shown that H_2_O_2_ is greatly increased in the exhaled breath condensate of COPD subjects compared to healthy controls [43,44] with exacerbating subjects levels of H_2_O_2_ increased even further. Arachidonic acid, an abundant polyunsaturated fatty acid in cell membranes, can be peroxidised by free radicals in vivo, to form isoprostanes [45], which can be measured in EBC and have been found to be increased in COPD [46]. This study was well matched for age and pack year history, but not for medication use (in particular, β2-agonist and steroid use) and previous research indicates that both medications can lower 8-isoprostane in EBC [47,48]. A further product of fatty acid peroxidation, malondialdehyde (MDA), was also found to be significantly higher in the EBC of 73 COPD subjects when compared to healthy controls, asthmatics and bronchiectatic subjects. The same study also showed that COPD MDA levels correlated inversely with forced expiratory volume in 1 second (FEV_1_) [49], suggesting a link with severity.

Serum levels of MDA and GPx (determined by activity) correlate with COPD severity [50,51], with increased serum MDA and reduced GPx correlating with increased COPD severity compared with healthy non-smokers. 

Using immunohistological staining, it is possible to visualise some products of oxidative stress within distinct cellular components of the lung, such as 4-hydroxy-2-nonenal (4HNE), an end product of lipid peroxidation, which readily forms adducts with several protein residues. The markers of nitrogen derived oxidative stress, nitrotyrosine and inducible nitric oxide synthase (iNOS) have been found to be increased in COPD [52] correlating inversely with FEV_1_ [53]. 

## 6. The Cellular Sources of ROS in COPD

### 6.1. Neutrophils

Neutrophils are thought to be key effector cells in COPD. Their numbers in COPD lungs are significantly increased [54], correlate with disease severity and their products have been shown to cause all pathological aspects of disease (including airflow obstruction, chronic bronchitis and emphysema) in animal and cell based models [55]. There is growing evidence that they display an aberrant phenotype compared to healthy controls that might favour tissue damage including increased speed, reduced accuracy during migration [56] coupled with increased markers of activation and degranulation (cell polarisation, CD63 expression) [57], and evidence of constituently active phosphoinositide 3 kinase (PI3K) [58] which has been associated with increased inflammation and proteinase activity measureable systemically [56]. Additionally, neutrophils from COPD patients have been shown to release increased amounts of ROS spontaneously [59] and following stimulation [59,60,61].

These altered functions provide a possible mechanism for the damage seen in COPD; as neutrophils migrate through lung tissues they are known to release proteinases and ROS in sequence as they move through complex tissues. Inaccurate migration may result in moving across a larger surface area, whilst undergoing increased degranulation. Products of degranulation include pro-inflammatory cytokines such as interleukin 8 (IL8), ROS and proteases such as neutrophil elastase (all increased in COPD sputum [62]) increasing inflammatory signalling and protease burden in the lung tissue across a wider area.

Of note, migration by neutrophils is known to be affected by ROS, which interact with the enzyme PTEN at the leading edge, reducing its activity [34], allowing the accumulation of phosphatidylinositol (3,4,5)-triphosphate, a molecule key in neutrophil migration, therefore excess intracellular ROS may alter migratory accuracy. Furthermore, PI3K inhibitors have been shown to reduce ROS release from COPD neutrophils, demonstrating the close associations between intracellular ROS activity and vital cell functions [63].

There are also important associations between neutrophil proteinases and ROS. Neutrophil elastase, released from primary granules during neutrophil activation, can digest many of the proteins in the extra cellular matrix [64], contribute to epithelial cell apoptosis [65] and induce mucus hypersecretion in goblet cells [66]. Neutrophil elastase is inhibited in a 1:1 molar complex by α1AT [67], an anti-proteinase. α1AT is able to reduce collateral damage to tissues during inflammation but not prevent it entirely, a short window of very high concentration immediately after release permits an area of obligate tissue damage before complete inhibition by α1AT [68]. α1AT also has important non-proteinase functions, such as a reduction in the active form of the pro-inflammatory cytokine TNF-α [69] and reducing apoptosis [70]. ROS are able to inactivate α1AT via oxidation of the methionine 358 residue in the active site [27,71], promoting inflammation. 

### 6.2. Monocytes/Macrophages

In health, macrophages are the most common immune cell found in the peripheral lung [72], where they will regularly encounter environmental ROS, and are key in the resolution of inflammation via efferocytosis of apoptotic neutrophils. Whilst there is little evidence that COPD macrophages produce abnormal amounts of ROS directly, there is evidence that pro-inflammatory cytokine [73] and enzyme [10] release is increased, whilst the macrophages display reduced intracellular thiol concentration, a marker of oxidative stress [74]. Activation of tissue resident macrophages results in the expression of chemokines, to recruit other immune cells. In COPD, there is evidence of macrophage activation, with increased expression of monocyte chemoattractant protein 1 (MCP-1) on the COPD bronchiolar epithelium and receptor CCR2 on COPD macrophages [75], resulting in increased recruitment of monocytes, correlating with disease severity [76]. Macrophages isolated from COPD patients have been associated with increased elastin degradation in vitro, producing increased amounts of matrix metalloproteinase (MMP) 9 [10], an enzyme with potent extracellular matrix activity. 

Once recruited, differentiation of monocytes is influenced by environmental conditions and stimuli, typical pro-inflammatory cytokines such as bacterial fragments and interferon-γ encourage differentiation into M1 phenotype, often referred to as “classical” or pro-inflammatory, as opposed to anti-inflammatory M2 phenotype. In COPD, there is evidence of altered monocyte and monocyte derived macrophage function, including reduced phagocytosis [77] and an increased inflammatory cytokine output (IL1β and IL12) [78]. Other research has described reduced M1 markers [79,80], and increased pro-inflammatory output [52,80].

An element of M2 macrophage anti-inflammatory phenotype is the secretion of the cytokine interleukin(IL)-10. This has an anti-inflammatory effect on other monocytes by inducing the intracellular protein histone deacetylase(HDAC)2 to inhibit the transcription of the pro-inflammatory cytokine IL-8. Interestingly, Ito et al. [81] have shown reduced expression of HDAC2 protein that correlates with COPD severity. In addition, it has been shown that neutrophil elastase (NE) can cleave the phosphatidylserine receptor [82], required for macrophage recognition of apoptotic cells, suggesting a possible reason for the reduced efferocytosis seen in COPD macrophages, and resultant pro-inflammatory environment.

### 6.3. Lung Epithelial Cells

Epithelial cells within the lung are likely to have an impact upon COPD progression, whilst there is little evidence of abnormal ROS production from the cells directly, lung epithelial cells are capable of contributing to the pro-inflammatory environment that is key to ROS production. Exposure to oxidative stress results in the release of pro-inflammatory cytokines [83,84], whilst continued exposure leads to alveolar epithelial cell necrosis [85], however, this has only been shown in vitro, using high doses of H_2_O_2_ and physiological in vivo antioxidants were absent. This has been linked to oxidation of an essential part of the caspase apoptosis pathway [86], caspase 3, which is inactivated in oxidative stress conditions [87,88], providing a potential pathway for increased cell necrosis and therefore inflammatory conditions within the lung. Hyperplasia of goblet cells within the lung epithelium leads to excess mucus production [89], and subsequently hypoxic conditions within bronchial fluids, leading to delayed apoptosis of neutrophils and subsequent pro-inflammatory mediator release.

## 7. Mechanisms by Which ROS May Lead to COPD Development or Progression

The functions of ROS in health can be linked logically to many features seen in COPD, as described in Table 1, however, these markers of oxidative stress may be caused by, and not causative of COPD. Most studies of COPD have focused on patients with moderate to severe disease, when structural damage is present and most inflammatory markers are increased [90]. However, there are some studies which have sought to determine the mechanisms by which ROS can cause the pathology seen in COPD.

One common model is chronic exposure of mice to ozone, which results in the display of COPD-like pathological changes such as lung inflammation, airway hyper-responsiveness, and neutrophil and macrophage infiltration of the lung. Using these models, research has shown that various anti-inflammatory or antioxidant molecules [91,92,93] have the ability to reduce inflammation and severity of COPD symptoms in the mouse model. Other murine models include exposure to cigarette smoke. Sato et al. [94] induced bronchiolar epithelial injury, emphysema, lung neutrophil and macrophage infiltration, increased oxidative stress markers and pro-inflammatory cytokines, through chronic exposure of mice to cigarette smoke (6 months). Transgenic mice expressing human thioredoxin-1 (TRX), an antioxidant molecule, displayed a reduction in many of the COPD-like changes that this treatment induced. Both of the above models used exposures to ROS that are greatly increased compared to levels that would be seen in vivo in humans, limiting their use as COPD models, however, they do provide insight into possible mechanisms that may be important in therapy.

Abnormal mitochondrial function related to ROS has recently been described in a number of studies supporting the hypothetical link between mitochondrial dysfunction and COPD. Wiegman et al. [92] studied airway smooth muscle (ASM) cells of COPD patients and mouse models of COPD. Introducing oxidative stress via H_2_O_2_ induced mitochondrial dysfunction in healthy ASM but did not worsen COPD dysfunction. Mitochondrial targeted antioxidant treatment inhibited mitochondrial dysfunction in healthy patients, and reduced excessive proliferation and cytokine production of ASM cells isolated from COPD patients with moderate to severe disease. Furthermore, Belchamber et al. [95] found evidence of mitochondrial dysfunction in COPD macrophages during phagocytosis and exacerbation, additional studies supporting oxidative stress induced mitochondrial dysfunction [96,97] suggest that this may be a promising field of research.

## 8. Why Might ROS Damage Be Heightened in COPD?

Markers of oxidative stress appear increased in COPD patients and it has been hypothesised that there may be an imbalance between oxidants and antioxidants in COPD that would contribute to disease pathogenesis and progression.

### 8.1. Reduced Anti-Inflammatory Defence in COPD

Some studies report reduced GSH in induced sputum of stable COPD subjects when compared to age matched healthy smokers and non-smokers, this level was even lower in COPD subjects who were exacerbating [51]. However, contrasting results have been reported by other investigators [103] who describe significantly lower GSH levels in bronchiolar lavage fluid (BAL) of non-smokers than in smokers and stable COPD. 

Some studies have suggested that mutations in SOD genetics may have an impact upon COPD incidence or pathogenesis. Siedlinski et al. [104] found a single nucleotide polymorphism (SNP) at C5774T in SOD2 led to an increase in risk of COPD, although the functional implications of this have not been described. In the same study, a mutation at 213 Gly in SOD3 was associated with a slower FEV_1_ decline in COPD. In vitro studies suggest that the 213 Gly SNP infers a resistance to post translational cleavage by furin proteases, which permits an extended half-life to SOD3, increasing tissue levels nine-fold or more [105,106] and allowing it to diffuse through tissues [107]. Dahl et al. [108] described two polymorphisms in SOD3 which were significantly associated with lower FEV_1_ and FVC, markers of disease severity, independent of the SOD3 213Gly variant. These polymorphisms occurred in exon 1 and intron 1, occurring with complete linkage, but were not deleterious, as tissue levels of SOD3 remain similar as in health.

Catalase activity has been shown to be significantly reduced in COPD sufferers compared to smokers and non-COPD subjects [98,99]. Betsuyaku et al. [100] found that immunohistochemical staining for catalase in bronchiolar epithelial cells was significantly decreased in smoking COPD subjects compared to smoking non-COPD subjects and non-smoking controls. There is some evidence expression is reduced in bronchiolar epithelium in COPD sufferers [100] and peripheral lung tissues [75], offering increased opportunity for oxidative stress conditions.

Some studies have described reduced levels of glutathione peroxidase in plasma of COPD patients [109], and levels correlate significantly with FEV_1_ [109]. However, this has not been replicated in studies of transcription [75], and the reported decreased GPx activity in blood [50,101] supports either a reduction in total GPx or a reduction in efficacy, reducing antioxidant function.

### 8.2. Increased Pro-Inflammatory Enzyme Expression in COPD

Studies of peripheral lung expression of inducible nitric oxide synthase (iNOS) (an enzyme responsible for creating ·NO) have reported no difference in iNOS expression in alveolar macrophages, however, type 2 pneumocytes expressing iNOS were more numerous in severe COPD [102], suggesting a higher ROS production and burden.

## 9. Therapeutic Studies

Currently, there are no clinically available treatments that prevent COPD progression. Studies of ROS related damage in COPD have been observational or described using cell and animal models, and therefore cannot prove causality. To further investigate the role of ROS in COPD pathogenesis, researchers have instigated clinical trials of antioxidants to determine efficacy on characteristic disease features. 

### 9.1. N-Acetyl-Cysteine (NAC)

NAC in vivo is rapidly metabolised to cysteine, where the thiol group is capable of reducing oxidant activity, reducing disulphide bonds and becoming a substrate for the formation of new GSH molecules [110]. NAC is also a mucolytic due to its ability to reduce disulphide bonds. 

The BRONCUS trial [111] treated 523 COPD patients with 600 mg NAC daily or a placebo and followed them for 3 years. FEV_1_ decline and exacerbation frequency was not improved by NAC treatment, however in a subset of patients not using inhaled corticosteroid, risk of exacerbation was lowered. Secondary analysis found that functional residual capacity was significantly reduced in the NAC group but the authors concluded that NAC was ineffective at this dose in COPD. The PANTHEON trial [112] assessed the exacerbation rate via patient self-reporting over one year using a higher dose of NAC (600 mg twice daily) and reported a significant reduction in acute exacerbations but were unable to identify whether this was due to the mucolytic properties of NAC or more general anti-oxidant activity.

A recent meta-analysis [113] of NAC treatment upon exacerbations in chronic bronchitis or COPD included 13 studies, covering 4155 patients in total and described a significant reduction. This suggests that NAC therapy may have a role in patients who experience these episodes, but that NAC does not impact on the defining feature of COPD (airflow obstruction).

### 9.2. Glutathione

Glutathione is an abundant antioxidant molecule but older clinical studies have shown mixed results using this therapeutically. Borok et al. [114] provided glutathione by aerosol in an open labelled study of 10 COPD patients, and detected a rise in epithelial lining fluid GSH and reduced superoxide release from macrophages, but could not assess clinical endpoints. 

GSH was not tolerated in a trial of eight asthmatic patients who experienced cough/breathlessness and a reduction in FEV1 of 19% [115] potentially due to sulphite formation in the nebulised solution, or the metabolism of glutathione into leukotriene B4 or C4, both potent pro-inflammatory bronchoconstrictors. 

### 9.3. Nrf2 and Nrf2-Activators

Nuclear factor erythroid 2-related factor 2 (Nrf2) is a key transcriptional factor in the production of antioxidant molecules [116,117,118] and expression is thought to be reduced in COPD [119,120]. Deficiency of functional Nrf2 causes mice to be increasingly susceptible to oxidative stress [121,122], whilst pharmacological activation of Nrf2 offered protection [123].

A trial in COPD patients of the Nrf2 activating molecule sulforaphane improved the reduced phagocytosis of bacteria witnessed in alveolar macrophages from COPD patients [124]. Nrf2 activators may also have the added benefit of improving resistance to viral entry and replication in cells, potentially helping reduce COPD patient vulnerability to viral exacerbation [125]. 

### 9.4. Vitamins C and E

Vitamins C and E are thought to act as ROS scavengers, therefore therapeutic dosing may offer some reduction in oxidative stress. Lower plasma levels of vitamin C have been linked with increased incidence of COPD [126], whilst higher levels of both vitamin C and E in serum are linked with improved FEV_1_ (reviewed in [127]). Therapeutic use of either is less well documented, however. A large study by Agler et al. [128] supplemented 38,597 women with 600IU vitamin E every other day resulting in a 10% reduction of risk of COPD, whilst Rautalahti et al. [129] found no benefit of vitamin E supplementation on the symptoms of COPD in a 50 mg/day, 29,133 person trial. Furthermore, a Cochrane review of antioxidant supplements described increased mortality risk associated with vitamin E [130].

Overall, studies of anti-oxidants in COPD have been disappointing. This might reflect an inappropriately chosen medicant, or its dose or route; a lack of power for clinically based endpoints, or the use of the treatment in the wrong patient subset (too severe disease when damage is established and not amenable to change, those with a lack of exacerbations). Further studies are warranted, but enhanced mechanistic insights are needed in order to focus such studies appropriately, particularly in light of new studies highlighting the role of mitochondrial dysfunction in ROS related tissue damage.

## 10. Conclusions

There are sound theoretical reasons why increased ROS release or reduced ROS clearance might lead to the development or progression of COPD. There is an increased oxidant burden in smokers. The increased oxidant burden results from the 4700 chemical compounds and more than 10^15^ oxidants/free radicals contained in cigarette smoke and that many of these oxidants are relatively long-lived (reviewed in [131]). However, this stimulus alone cannot be sufficient or necessary to cause COPD in smokers, suggesting that there must be susceptibility factors which predispose to this condition.

Many products of oxidative stress have been shown to be increased in COPD compared with healthy smoking and non-smoking controls, whilst corresponding levels of enzymes responsible for removing ROS appear reduced in some studies. Cell studies suggest increased ROS release from key mediators of the inflammatory response in COPD including neutrophils, airway macrophages and monocytes. Although there is no animal model of COPD that replicates all clinical facets of disease, murine models have described increased oxidative burden following cigarette smoke exposure and subsequent tissue damage including the development of emphysema, which can be partially attenuated by targeting oxidation pathways. It is unclear whether the loss of balance between oxidants and anti-oxidants are a product of chronic inflammation seen in COPD or an initiating event. Specific oxidant/antioxidant genetic mutations have been associated with disease in humans, but these are relatively rare and could only account for a small proportion of cases.

The great heterogeneity in COPD, including clinical presentation and course as well as the variation in proteins, enzymes, molecules and cells involved in COPD are a significant challenge when looking for potential therapies. It is currently unclear whether over- or under-expression of oxidant/anti-oxidants is uniform amongst COPD sufferers, or whether sub populations exist. Understanding this is vital in order to stratify which groups may benefit most from anti-oxidant therapies. Clearly, further basic and translational research is needed to identify which patients become susceptible to ROS related damage and to clarify whether ROS is an effective target for change in COPD.

## Figures and Tables

**Table 1:** Potential relationships between features of COPD and functions of ROS.

Functions of ROS	Potentially Related Features of COPD
Alteration of biological molecules: Thiols [22] Amines [23] Amino acid residues [24] Charge profiles and disulphide bond formation [25,26] DNA/RNA [31]	Reduced antioxidant and antiprotease enzyme activity: SOD [98,99] Catalase [98,99] GPx [98] α1AT [27,28]
	Altered expression of ROS related enzymes: ↓ Catalase [75,100] ↓ GPx [50,101] ↓ SOD [98] ↑ iNOS [102]
Mitochondrial respiration [32]	Altered mitochondrial function [92,95,96,97]
Intracellular signalling [33,34]	Altered expression of ROS related enzymes: ↓ Catalase [75,100] ↓ GPx [50,101] ↓ SOD [98] ↑ iNOS [102]

Note: ROS, reactive oxygen species; COPD, chronic obstructive pulmonary disease; DNA, deoxyribonucleic acid; RNA, ribonucleic acid; SOD, superoxide dismutase; GPx, glutathione peroxidase; α1AT, α1 antitrypsin; iNOS, inducible nitric oxide synthase.
